# Patriotism and the Nurse-Training Schools

**Published:** 1916-11-18

**Authors:** 


### PATRIOTISM AND THE NURSE-TRAINING SCHOOLS.

### X. Leicester, Sheffield, Salisbury.

These noted training-schools for nurses are in centres where in normal times the civilian pressure on the accommodation is seldom relaxed. In all three cases many of the most highly skilled members of the nursing staff have been placed at the disposal of the military authorities, while the hospitals have undertaken much valuable work for the wounded.

### Leicester Royal Infirmary.

The matron of the Royal Infirmary is also principal matron of the 5th Northern General Hospital Unit, comprising some 2,000 beds, which is established at Leicester. At the outbreak of war six members of the nursing staff and many of the infirmary sisters were called up on the mobilisation of the Territorial Force Nursing Service. The following members of the nursing staff are on active service abroad either in France, in the East, or on ship transport work:—

Sisters Sawyer, Sumner, Conway-Jones, Trotter, Lynch, and Dodds; Nurses Hunter, Cameron, Addison, Beardsley, Davies, Oakley, Watmore, Workman, Willey, E. Watson, Cole, and Hunt. Also Sisters Fisher, Roberts, Burlend, and Seacome, and Nurse Harvey on foreign service.

The following are on service in home military hospitals at Millbank, Haslar, etc.:—

Sisters Bates and A. Simpson, Nurses May, Ford, and Watherstone.

Among those trained at Leicester Royal Infirmary on war duty, the following are working in the T.F.N.S. at the 5th Northern General Hospital and the North Evington War Hospital, Leicester:—

Nurses Barnes, Birch, Eacott, Fussell, Glenn, I. German, Knowles, Perry, Potter, Roberts, Sly, Seacombe, Willis, Pare, Archer, Andrews, Cameron, Clark, Cartwright, Davis, Fiddler, Grocock, Hart, Holroyd, Highley, Harvey, Mairs, N. Simpson, H. Simpson, Wren, Taylor, N. Harries, R. Oliver, and Miss Parsons, late sister of the Royal Infirmary.

Four wards, comprising 162 beds, at the Royal Infirmary have been given over to the War Office for the wounded, and the following infirmary sisters are working in connection with these military wards:—Sisters Crosskill, Mills, Hone, Francis, Rayner, and Embry.

### Sheffield Royal Hospital.

Miss Earle, the matron of this hospital, is also matron of the 3rd Northern General Hospital, and is now on war duty. At the commencement of the war the board of management gave up 100 beds for the wounded soldiers, sixty of which are now in use, three wards of twenty beds each. The other forty beds are at the hospital annexe at Fulwood, and are being used for the convalescent wounded. During the first few months of the war about fifty members of the Red Cross Voluntary Aid Detachments were received into the hospital for a few hours daily to gain a little practical experience. During the past year nearly 200 men of the R.A.M.C. have been admitted to the hospital also from time to time for practical training. The following members of the staff are doing war work:—Sister Brown, in Egypt; Sisters Walker and Evans, in France; Sisters Franklin, Munstone, Peach, and Young, T.F.N.S.

Among late members of the nursing staff now on war service is Nurse M. Bannister, lately mentioned in dispatches for excellent work done in France. Others are Nurses D. Widdop, Wilson, and Creswell, in France; Nurses Seaton, B. Wilbairn, and Musgrave, in Egypt; Nurses Ryder and Legg, in Malta; Sisters Macer, Stevenson, and Astell, T.F.N.S.; and in the same service Nurses Nichol, Lawson, Gregory, Russell, Winter, Porter, Alexander, Martin, Parker, Proudlove, Gordon, Cutts, Tait, Grayson, Holford, Robertson, Hughes, Atkinson, C. Brown, Morgan, Banham, Bull, Thornborrow, Dickens, Vickers, and Crowder.

### Salisbury Infirmary.

When war broke out the following sisters were sent off at once in fulfilment of the promise made to the War Office:—Sister Amy Lee and Nurse M. Jones, to Egypt; Sisters Sykes and Jeffrey, to France. Of these Miss Jeffrey has returned after a year's service and is on duty once more in her own hospital. The following joined later:—Sister Brotherton went to France, Sister Kate Ward to home service, Nurse Jupe to Egypt, and Nurse Chaplin to France. *T.F.N.S.*—Sister W. M. B. Friend (who has received the Royal Red Cross), to Egypt. Sisters M. Cressy, W. Rust, G. Faulkner, and E. Jackman for home service. Nurses Moore, Hearn, Elliott, Roberts, Milborne, Barfoot, and Watts have all left for service in Q.A.I.M.N.S.R., Nurse Watts having orders to go to the Mediterranean.

Many others who had completed their training at Salisbury are now members of Q.A.I.M.N.S. or of the T.F.N.S., but exact particulars are not available. Several of these are on active service. Enough has been said to give a very forcible impression of the extent to which the war necessitated reorganisation among the nursing staff of the infirmary. The greatest credit is due to those members of the staff who have creditably carried on the work at great pressure in the absence of so many heads of departments.

